# Supplementary material for: The Non-Fibrillating N-Terminal of α-Synuclein Binds and Co-Fibrillates with Heparin
Source: Biomolecules. 2020 Aug 16;10(8):1192. doi: 10.3390/biom10081192 (PMC7464290; doi:10.3390/biom10081192)
Supplement: Supplementary file 1 [file biomolecules-10-01192-s001.pdf]

SUPPORTING FIGURES AND TABLES

The Non-Fibrillating N-terminal of α-Synuclein Binds and Co-Fibrillates with Heparin

Line K. Skaanning[1], Angelo Santoro[2,3], Thomas Skamris[1], Jacob Hertz Martinsen[1,2], Anna Maria D'Ursi[3], Saskia Bucciarelli[1], Bente Vestergaard[1], Katrine Bugge[2], Annette E. Langkilde[1,#], Birthe B. Kragelund[2,#]

SUPPORTING FIGURES

Figure S1 – Purification of His-SUMO-aSN$_{1\text{-}61}$.

Figure S2 - SAXS data of aSN$_{1\text{-}61}$ at different concentration.

Figure S3 – Test of fibrillation properties for aSN$_{1\text{-}61}$.

Figure S4 – ThT control measurement.

Figure S5 – Normalized residual concentration in supernatants after fibrillation

Figure S6 - SAXS data of heparin in different buffers with different salt concentrations

Figure S7 – ThT and CD analyses of factors potentially affecting aSN$_{1\text{-}61}$ fibrillation

SUPPORTING TABLES

Table S1 – Primary analysis of aSN$_{1\text{-}61}$ SAXS data as shown in Figure S2A

Table S2 - Primary analysis of SAXS data for heparin (Figure S7).

Table S3 – Overview of fragments of aSN, and their fibrillation/aggregation potential reported from the literature

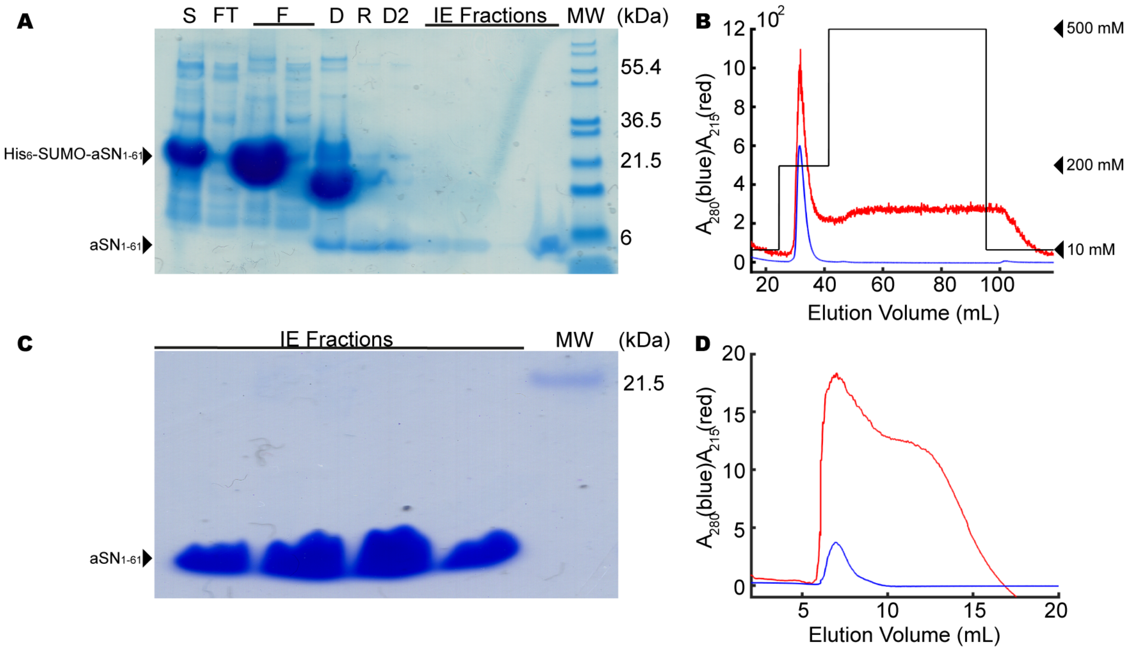

Figure S1 – Purification of His-SUMO-aSN$_{1\text{-}61}$. A) SDS-PAGE of fractions from the initial steps in purification of aSN$_{1\text{-}61}$. Stained with InstantBlue™ . S: Supernatant cell lysis. FT: flow-through from HisTrap step. F: Fractions from HisTrap. D: Dialysate of cleaved with His-ULP1. R: Pooled fraction 3-13 from reverse HisTrap D2: dialysate of aSN$_{1\text{-}61}$ into 50 mM Tris, 10 mM NaCl, pH 8.5. IE: Fractions from ion exchange. B) Chromatogram showing the stepwise elution profile of His-SUMO-aSN$_{1\text{-}61}$ on the HisTrap column. Blue trace corresponds to absorption at 280 nm and red trace corresponds to absorption at 215 nm. C) SDS-PAGE of the fractions from ion exchange of purification of aSN$_{1\text{-}61}$. The gel was stained with Coomassie. D) Elution profile of aSN$_{1\text{-}61}$ from IE column. Blue trace corresponds to absorption at 280 nm and red trace corresponds to absorption at 215 nm. The base line for absorbance at 280 nm and 215 nm was set to overlap in the beginning of both chromatograms.

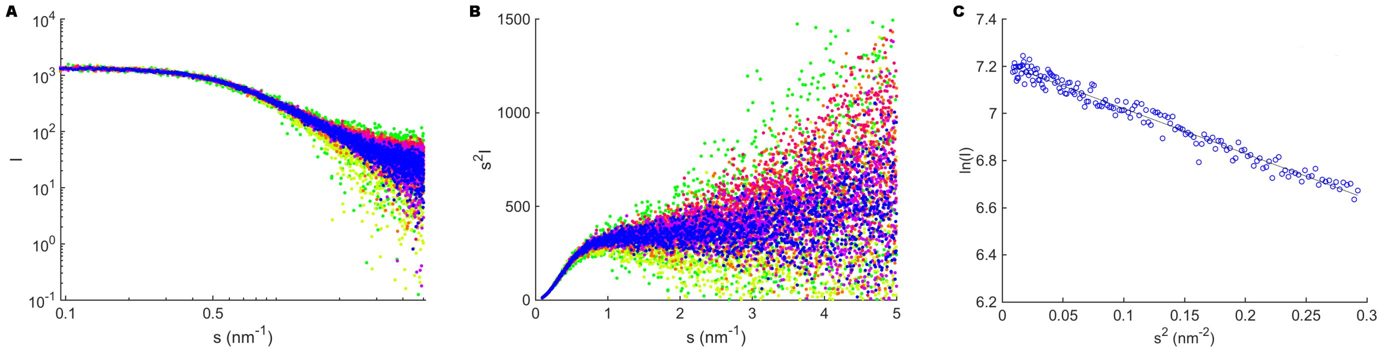

Figure S2 - SAXS data of aSN_{1-61} at different concentration. A) Scattering intensities as a function of the momentum transfer. Scattering curves were initially scaled by concentration and final adjustments made by scaling to the highest concentration sample. 223 µM (light green), 287 µM (lime), 366 µM (orange), 446 µM (magenta), 510 µM (purple) and 573 µM (bluu) and B) corresponding Kratky plot, all samples shown (colors as in panel A). C) Guinier range (experimental data in blue) and fit (black) for aSN_{1-61} at 573 µM.

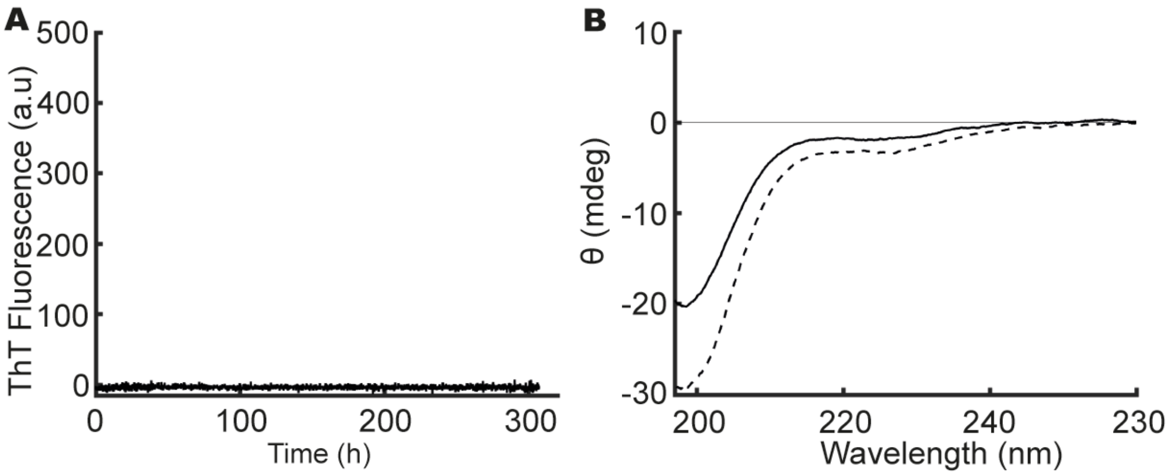

Figure S3 – Test of fibrillation properties for aSN_{1-61}. A) ThT profile of aSN_{1-61} during incubation in conditions known to induce fibrillation of aSN. B) Far-UV CD spectrum before incubation (solid) and after incubation (dashed).

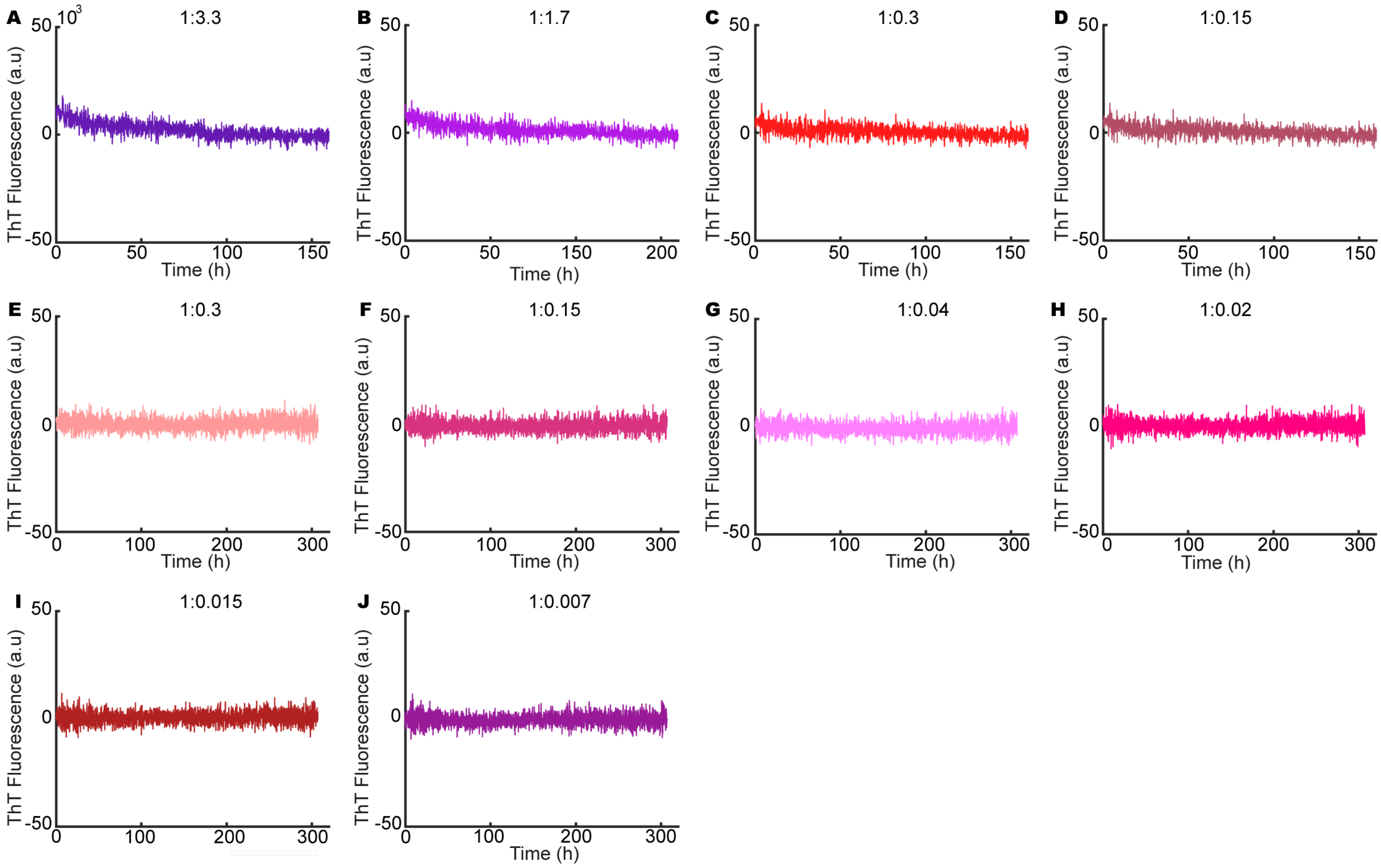

Figure S4 – ThT control measurement. Fluorescence emission at 480±5 nm (ThT assays) of heparin at concentrations corresponding to those used in the experiments with incubation of aSN$_{1\text{-}61}$ and heparin (Figures 2 and 3). The ratios above each panel indicate the corresponding experiments with aSN$_{1\text{-}61}$. The specific heparin concentrations are: A) 666 µM heparin; B) 333 µM heparin; C) 66.7 µM heparin; D) 29.3 µM heparin; E) 29.3 µM heparin; F) 14.7 µM heparin; G) 3.7 µM heparin; H) 1.8 µM heparin; I) 1.5 µM heparin; J) 0.7 µM heparin. The concentration for heparin in D and E is the same, as the aSN$_{1\text{-}61}$:heparin experiments were performed with different aSN$_{1\text{-}61}$ concentrations.

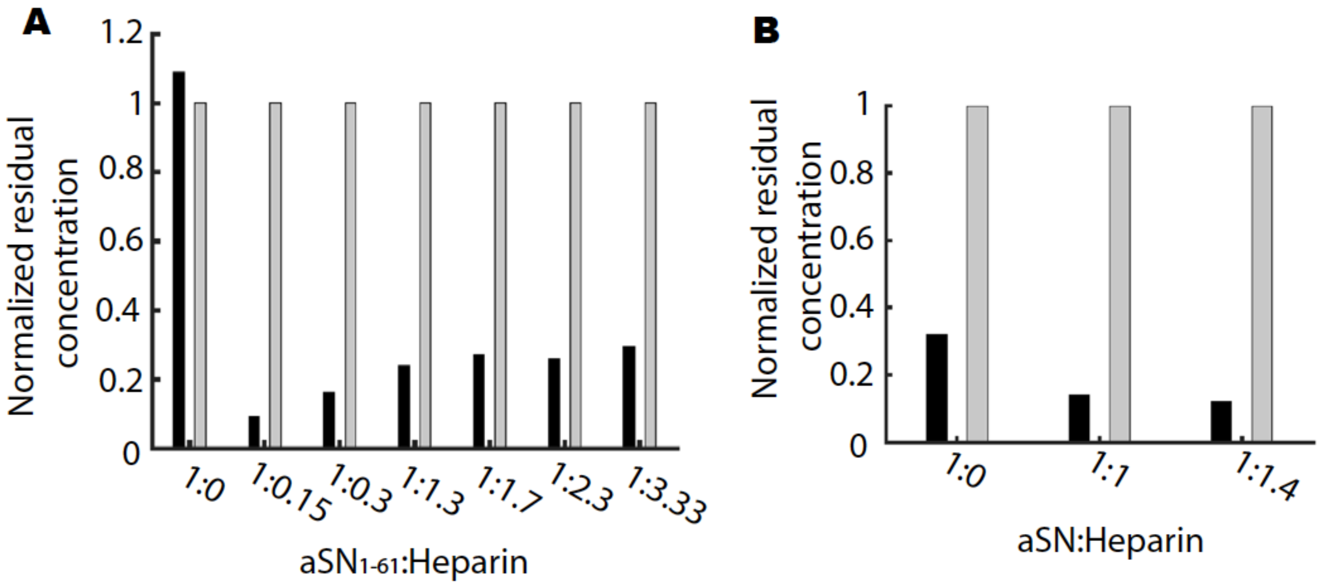

Figure S5 – Normalized residual concentration of (A) aSN$_{1\text{-}61}$ and (B) aSN in the supernatant after incubation (black) and before incubation (grey) with different molar ratios of (A) aSN$_{1\text{-}61}$:heparin and (B) aSN:heparin.

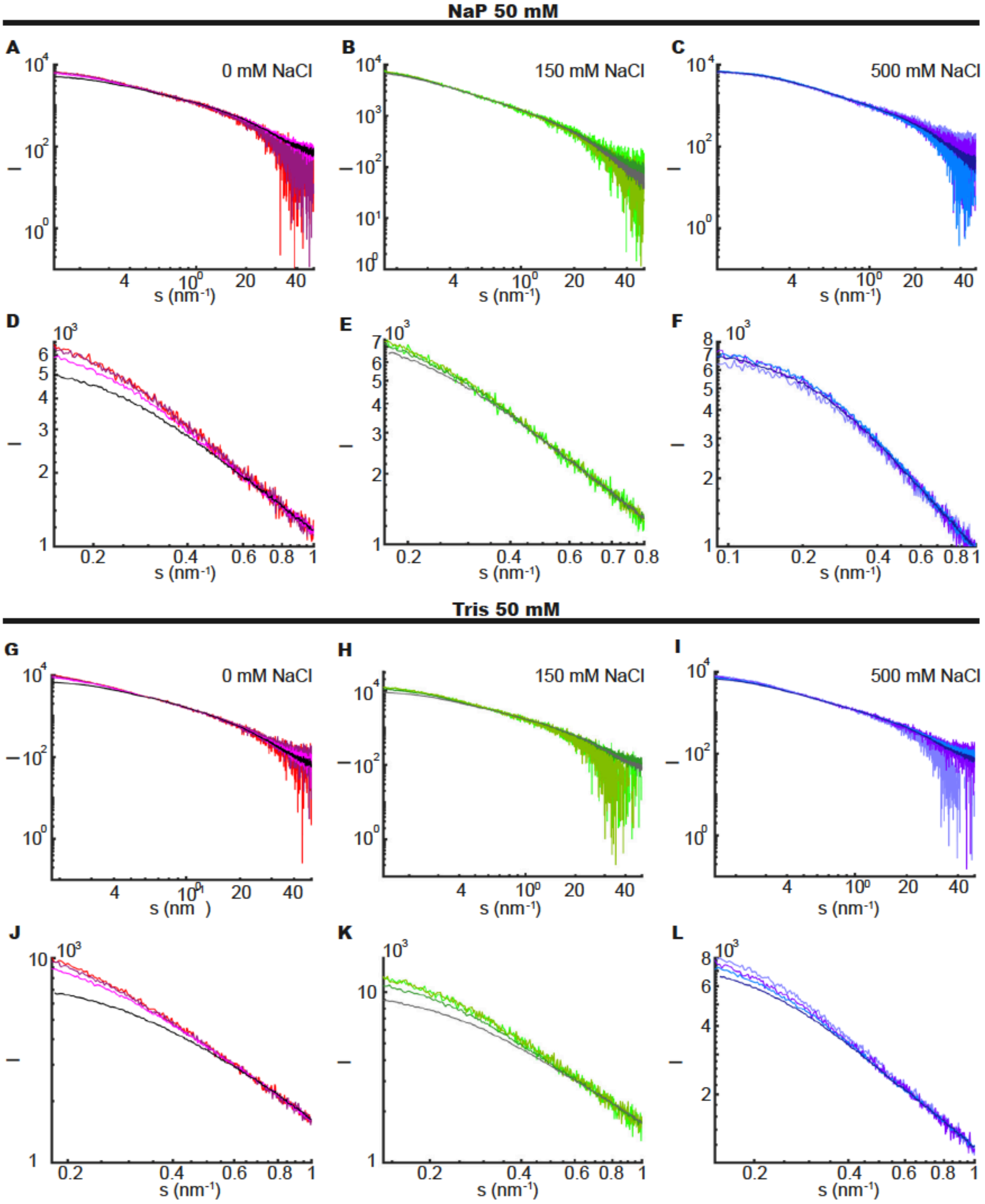

Figure S6 - SAXS data of heparin in different buffers with different salt concentrations. All data are shown on absolute scale and normalized by concentration. A) heparin in 50 mM NaP 0 mM NaCl. Concentration of heparin 53.3 µM (red), 66.7 µM (purple), 133 µM (magenta), 333 µM (black). B) heparin in 50 mM NaP 150 mM NaCl. Concentration of heparin 53.3 µM (light green), 66.7 µM (green), 133 µM (dark green), 333 µM (grey). C) Heparin in 50 mM NaP 500 mM NaCl. Concentration of heparin 53.3 µM (light blue), 66.7 µM (light purple), 133 µM (dark purple), 333 µM (dark blue). D-F) Zoom of low-s region of A-C, respectively. G) heparin in 50 mM Tris, 0 mM

NaCl. Concentration of heparin 53.3 µM (red), 66.7 µM (purple), 133 µM (magenta), 333 µM L (black). H) heparin in 50 mM Tris 150 mM NaCl. Concentration of heparin 53.3 µM (light green), 66.7 µM (green), 133 µM (dark green), 333 µM (grey). I) Heparin in 50 mM Tris 500 mM NaCl. Concentration of heparin 53.3 µM (light blue), 66.7 µM (light purple), 133 µM (dark purple), 333 µM (dark blue). J-L) Zoom of low-s region of G-I, respectively

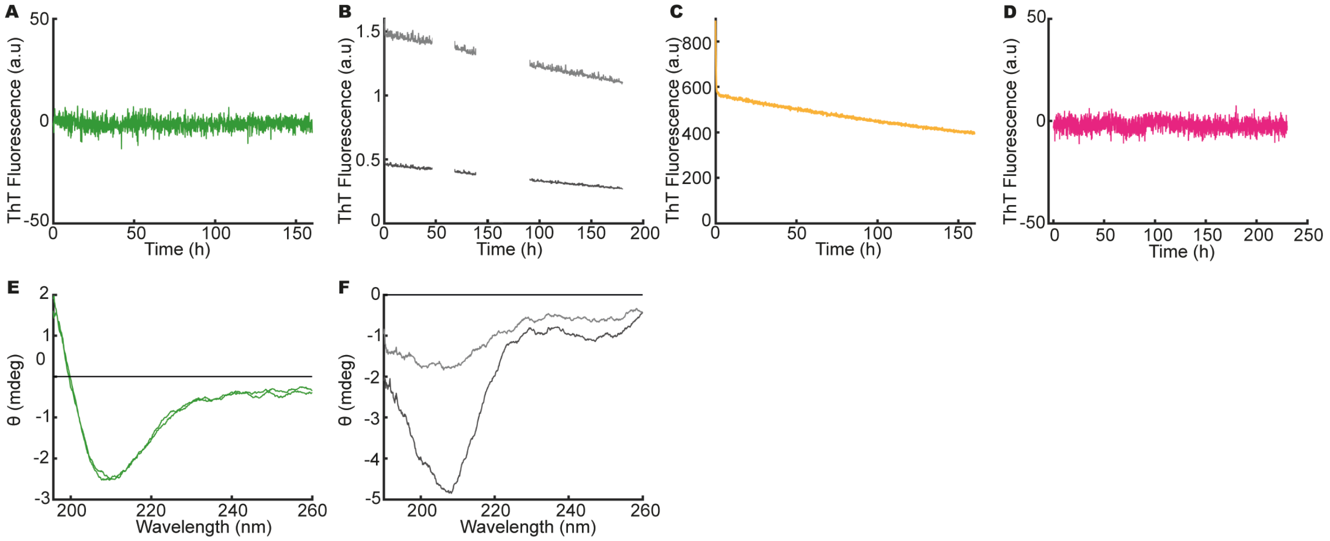

Figure S7 – ThT and CD analyses of factors potentially affecting aSN_{1-61} fibrillation. A) ThT assay of heparin alone incubated with 1 M NaCl. B) ThT assay of CS alone at 3 mg/mL (light grey) and 0.9 mg/mL (dark grey). C) ThT assay of β-casein at 333 µM. D) ThT assay of aSN_{1-61} incubated in 160 mM ammonium sulfate. E) Far-UV CD spectrum of heparin (green) in 1 M NaCl before (solid) and after (dotted) fibrillation. F) Far-UV CD spectrum of CS at 3 mg/mL (light grey) and 0.9 mg/mL (dark grey) after fibrillation.

Table S1 – Primary analysis of aSN$_{1\text{-}61}$ SAXS data as shown in Figure S2A. The apparent values of R_g and I_0 as obtained from Guinier analysis in PRIMUS [1] together with estimated molecular weights based on the Bayesian inference approach [2].

Sample conc (µM)	R_g (Å)	I_0 relative scale	MW estimate (credibility interval) (kDa)
573	24.0±0.3	1360±5	11.3 (10.0-12.4)
510	24.1±0.4	1358±5	11.2 (10.0-12.4)
446	24.2±0.8	1367±5	9.5 (9.1-12.4)
366	24.8±1.6	1390±6	11.2 (10.0-12.4)
287	24.8±0.6	1409±7	15.5 (14.5-16.5)
223	25.1±1.7	1407±10	11.2 (10.0-12.4)

Table S2 - Primary analysis of SAXS data for heparin (Figure S7). Heparin in 50 mM NaP with 0, 150 or 500 mM NaCl (top) and heparin in 50 mM Tris with 0, 150, or 500 mM NaCl (bottom). The apparent values of R_g and I_0 (i.e. with no correction for intermolecular interactions) are shown together with the apparent estimated molecular weight based on absolute scaling to illustrate the systematic influence of the interparticle interaction effects.

Heparin	50 mM NaP 0 mM NaCl			50 mM NaP 150 mM NaCl			50 mM NaP 500 mM NaCl		
(µM)	R_g (nm)	I_0	MW (kDa)	R_g (nm)	I_0	MW (kDa)	R_g (nm)	I_0	MW (kDa)
333	3.91±0.43	5549±16	5.5	4.17±1.62	7815±32	7.8	5.19±0.48	7386±19	7.4
133	4.53±0.6	6885±31	6.9	4.88±0-15	9038±39	9	4.72±0.09	7406±27	7.4
66.7	4.73±0.49	7588±47	7.6	5.08±0.23	9597±54	9.6	4.77±0.7	7296±54	7.3
53.3	4.78±0.78	7734±57	7.7	5.08±0.33	9578±89	9.6	4.67±0.32	6795±45	6.8

Heparin	50 mM Tris 0 mM NaCl			50 mM Tris 150 mM NaCl			50 mM Tris 500 mM NaCl		
(µM)	R_g (nm)	I_0	MW (kDa)	R_g (nm)	I_0	MW (kDa)	R_g (nm)	I_0	MW (kDa)
333	3.37±0.97	7255±14	7.2	4.5±1.8	10263±27	10.3	4.48±0.78	10431±33	10.4
133	4.74±0.35	11042±35	11	4.8±0.25	907±40	9	4.91±0.86	12311±48	12.3
66.7	5.14±0.67	12885±51	12.9	5.07±0.36	5602±44	5.6	5.30±0.47	9498±52	9.5
53.3	5.25±0.34	12562±56	12.6	5.29±0.47	7051±59	7	5.21±0.51	13950±70	13.9

Table S3 – Overview of fragments of aSN, and their fibrillation/aggregation potential reported from the literature.

Fragment	Observation	Reference
1-60	No fibrillation<br>Fibrillation in trifluoroethanol	[3,4]
1-65	Aggregation	[5]
1-70	Aggregation	[3]
1-87	Fibrillation	[6]
1-95	Aggregation	[5]
1-100	Fibrillation<br>Fibrillation in trifluoroethanol	[3,4]
1-108	Fibrillation	[7]
1-110	Fibrillation	[8] [9]
1-120	Fibrillation	[6,8–10]
1-124	Fibrillation	[7]
1-130	Fibrillation	[9]
11-140	Fibrillation	[11]
15-95	Aggregation	[5]
15-65	Aggregation	[5]
16-35	Fibrillation	[5]
21-140	Fibrillation	[11]
31-140	Fibrillation	[11]
32-140	Fibrillation	[10]
41-140	Fibrillation	[11]
51-140	Fibrillation	[11]
58-140	Aggregation	[10]
60-95	Fibrillation	[12]
NAC 1-18 (61-78)	Aggregation	[13]

NAC8-15 (68-75)	No fibrillation	[13]
NAC8-16 (68-76)	No fibrillation	[13]
NAC8-18 (68-78)	Fibrillation	[13]
NAC9-16 (69-76)	No fibrillation	[13]
71-140	No fibrillation	[11]
NAC12-18 (72-78)	No fibrillation	[13]
NAC 19-35 (79-95)	No fibrillation	[13]
93-115	Fibrillation	[5]
116-136	Fibrillation	[5]
Δ71-82	No fibrillation	[14]
Δ71-74	Fibrillation	[3]
Δ66-74	Fibrillation	[3]
Δ1-74	Fibrillation	[3]
Δ71-82	Fibrillation	[11]
Δ73-83	Fibrillation	[11]

REFERENCES

(1) Konarev, P. V; Volkov, V. V; Sokolova, A. V; Koch, H. J.; Svergun, D. I. PRIMUS : A Windows PC-Based System for Small- Angle Scattering Data Analysis. *J. Appl. Crystallogr.* **2003**, *36*, 1277–1282.

(2) Hajizadeh, N. R.; Franke, D.; Jeffries, C. M.; Svergun, D. I. Consensus Bayesian Assessment of Protein Molecular Mass from Solution X-Ray Scattering Data. *Sci. Rep.* **2018**, *8* (1), 7204. https://doi.org/10.1038/s41598-018-25355-2.

(3) Du, H. N.; Tang, L.; Luo, X. Y.; Li, H. T.; Hu, J.; Zhou, J. W.; Hu, H. Y. A Peptide Motif Consisting of Glycine, Alanine, and Valine Is Required for the Fibrillization and Cytotoxicity of Human α-Synuclein. *Biochemistry* **2003**, *42* (29), 8870–8878. https://doi.org/10.1021/bi034028+.

(4) Li, H. T.; Du, H. N.; Tang, L.; Hu, J.; Hu, H. Y. Structural Transformation and Aggregation of Human α-Synuclein in Trifluoroethanol: Non-Amyloid Component Sequence Is Essential and β-Sheet Formation Is Prerequisite to Aggregation. *Biopolymers* **2002**, *64* (4), 221–226. https://doi.org/10.1002/bip.10179.

(5) Shen, N.; Song, G.; Yang, H.; Lin, X.; Brown, B.; Hong, Y.; Cai, J.; Cao, C. Identifying the Pathological Domain of Alpha-Synuclein as a Therapeutic for Parkinson's Disease. *Int. J. Mol. Sci.* **2019**, *20* (9), 1–16. https://doi.org/10.3390/ijms20092338.

(6) Serpell, L. C.; Berriman, J.; Jakes, R.; Goedert, M.; Crowther, R. A. Fiber Diffraction of Synthetic α-Synuclein Filaments Shows Amyloid-like Cross-β Conformation. *Proc. Natl. Acad. Sci. U. S. A.* **2000**, *97* (9), 4897–4902. https://doi.org/10.1073/pnas.97.9.4897.

(7) Hoyer, W.; Antony, T.; Cherny, D.; Heim, G.; Jovin, T. M.; Subramaniam, V. Dependence of α-Synuclein Aggregate Morphology on Solution Conditions. *J. Mol. Biol.* **2002**, *322* (2), 383–393. https://doi.org/10.1016/S0022-2836(02)00775-1.

(8) Liu, C. W.; Giasson, B. I.; Lewis, K. A.; Lee, V. M.; DeMartino, G. N.; Thomas, P. J. A Precipitating Role for Truncated α-Synuclein and the Proteasome in α-Synuclein Aggregation: Implications for Pathogenesis of Parkinson Disease. *J. Biol. Chem.* **2005**, *280* (24), 22670–22678. https://doi.org/10.1074/jbc.M501508200.

(9) Crowther, R. A.; Jakes, R.; Spillantini, M. G.; Y, M. G. Synthetic Filaments Assembled from C-Terminally Truncated Alpha-Synuclein. *FEBS* **1998**, *436*, 309–312.

(10) Guo, J. L.; Covell, D. J.; Daniels, J. P.; Iba, M.; Stieber, A.; Zhang, B.; Riddle, D. M.; Kwong, L. K.; Xu, Y.; Trojanowski, J. Q.; Lee, V. M. Y. Distinct α-Synuclein Strains Differentially Promote Tau Inclusions in Neurons. *Cell* **2013**, *154* (1), 103–117. https://doi.org/10.1016/j.cell.2013.05.057.

(11) Zibaee, S.; Jakes, R.; Fraser, G.; Serpell, L. C.; Crowther, R. A.; Goedert, M. Sequence Determinants for Amyloid Fibrillogenesis of Human α-Synuclein. *J. Mol. Biol.* **2007**, *374* (2), 454–464. https://doi.org/10.1016/j.jmb.2007.09.039.

(12) Han, H.; Weinreb, P. H.; Lansbury, P. T. The Core Alzheimer's Peptide NAC Forms Amyloid Fibrils Which Seed and Are Seeded by β-Amyloid: Is NAC a Common Trigger or Target in Neurodegenerative Disease? *Chem. Biol.* **1995**, *2* (3), 163–169. https://doi.org/10.1016/1074-5521(95)90071-3.

(13) El-Agnaf, O. M. A.; Irvine, G. B. Aggregation and Neurotoxicity of α-Synuclein and Related Peptides. *Biochem. Soc. Trans.* **2002**, *30* (4), 559–565. https://doi.org/10.1042/BST0300559.

(14) Giasson, B. I.; Murray, I. V. J.; Trojanowski, J. Q.; Lee, V. M. Y. A Hydrophobic Stretch of 12 Amino Acid Residues in the Middle of α-Synuclein Is Essential for Filament Assembly. *J. Biol. Chem.* **2001**, *276* (4), 2380–2386. https://doi.org/10.1074/jbc.M008919200.
